# The Role of Mitochondria in Liver Ischemia-Reperfusion Injury: From Aspects of Mitochondrial Oxidative Stress, Mitochondrial Fission, Mitochondrial Membrane Permeable Transport Pore Formation, Mitophagy, and Mitochondria-Related Protective Measures

**DOI:** 10.1155/2021/6670579

**Published:** 2021-07-05

**Authors:** Haifeng Zhang, Qi Yan, Xuan Wang, Xin Chen, Ying Chen, Jian Du, Lijian Chen

**Affiliations:** ^1^Department of Clinical Medical, The First Clinical Medical College of Anhui Medical University, Hefei, Anhui 230032, China; ^2^Department of Biochemistry and Molecular Biology, School of Basic Medical Sciences, Anhui Medical University, Hefei 230032, China; ^3^Department of Anesthesiology, The First Affiliated Hospital of Anhui Medical University, China

## Abstract

Ischemia-reperfusion injury (IRI) has indeed been shown as a main complication of hepatectomy, liver transplantation, trauma, and hypovolemic shock. A large number of studies have confirmed that microvascular and parenchymal damage is mainly caused by reactive oxygen species (ROS), which is considered to be a major risk factor for IRI. Under normal conditions, ROS as a kind of by-product of cellular metabolism can be controlled at normal levels. However, when IRI occurs, mitochondrial oxidative phosphorylation is inhibited. In addition, oxidative respiratory chain damage leads to massive consumption of adenosine triphosphate (ATP) and large amounts of ROS. Additionally, mitochondrial dysfunction is involved in various organs and tissues in IRI. On the one hand, excessive free radicals induce mitochondrial damage, for instance, mitochondrial structure, number, function, and energy metabolism. On the other hand, the disorder of mitochondrial fusion and fission results in further reduction of the number of mitochondria so that it is not enough to clear excessive ROS, and mitochondrial structure changes to form mitochondrial membrane permeable transport pores (mPTPs), which leads to cell necrosis and apoptosis, organ failure, and metabolic dysfunction, increasing morbidity and mortality. According to the formation mechanism of IRI, various substances have been discovered or synthesized for specific targets and cell signaling pathways to inhibit or slow the damage of liver IRI to the body. Here, based on the development of this field, this review describes the role of mitochondria in liver IRI, from aspects of mitochondrial oxidative stress, mitochondrial fusion and fission, mPTP formation, and corresponding protective measures. Therefore, it may provide references for future clinical treatment and research.

## 1. Introduction

Liver ischemia-reperfusion injury (IRI) occurs when blood supply is interrupted or sharply reduced, which is the main complication of liver operation, such as hepatectomy, liver transplantation, and trauma [1, 2]. IRI has been reported to occur in a variety of organs: heart [3, 4], kidney [5], brain [6], and lung [7], which has high morbidity and mortality worldwide. As the largest internal organ of the human body, the liver is supplied by liver arteries and portal veins with powerful metabolic functions. Since the production of liver energy depends on the supply of oxygen and the function of mitochondria, thus under the condition of ischemia and hypoxia, the liver function is inhibited and damaged [8].

Usually, liver IRI is divided into two types: Warm IRI occurs in low blood flow states, such as portal vein embolism, cirrhosis, liver resection, and liver transplantation. In this process, the structure and function of mitochondria are damaged, leading to inhibition of oxidative phosphorylation and reduction of adenosine triphosphate (ATP) production and further induce mitochondrial fission, mitochondrial autophagy, and apoptosis [9]. Cold IRI occurs during cryopreservation before isolated liver transplantation. Low temperature can slow down cell metabolism and cell oxygen consumption, thereby maintaining the structure and function of mitochondria. Static cold storage has been used as the main storage method to reduce the liver's temperature and maintain liver activity [10, 11]. Compared with static cold storage, mechanical perfusion can improve graft function and survival rate, and the mechanical perfusion technology under normal temperature is also constantly developing and improving [12, 13].

A wide variety of studies have shown that the mechanism of IRI is complex and not very clear, which involves multiple pathways: oxidative stress, inflammatory response, mitochondrial dysfunction, apoptosis, etc. Of note, these processes begin during the ischemic phase and strengthen during the reperfusion phase [14–16]. Under normal circumstances, the generation and elimination of reactive oxygen species (ROS) are maintained in a dynamic range. However, when the content of ROS increases or the activity of ROS-scavenging enzymes decreases, the balance will be destroyed, and too much ROS will lead to the changes in the morphology and membrane permeability of mitochondria to damage the mitochondrial oxidative respiratory chain, which leads to the inhibition of oxidative phosphorylation and the disorder of system energy metabolism [17]. In addition, mitochondrial fission, autophagy, and mitochondrial membrane permeable transport pores (mPTPs) are important mechanisms of liver IRI formation. Moderate mitochondrial division can maintain the number of mitochondria so that they can perform better functions, while a large number of mitochondrial fission will lead to the formation of mitochondrial fragmentation, activate the apoptosis pathway, and aggravate IRI [18, 19]. Liver resection and liver transplantation lead to the occurrence of IRI, while the application of ischemic preconditioning (IPC) and some drugs could decrease liver damages [20].

Therefore, this review mainly introduces the mechanisms of mitochondria in IRI from the aspects of oxidative stress, mitochondrial fusion and fission, mPTP formation, and corresponding protective measures to provide references for future clinical treatment and research.

## 2. Mitochondrial Oxidative Stress

Under normal circumstances, O_2_^•−^ is the one-electron reduction product of O_2_ which directly or indirectly generates all ROS. ROS plays a dual role in ischemia-reperfusion. For one thing, ROS activates the antioxidant systems to clear excessive ROS and promotes the survival of hepatocytes under hypoxia conditions. For another, when ROS is produced so much that the body cannot clear it, the imbalance between the oxidation and antioxidant systems in the body leads to oxidative stress, which will lead to mitochondrial dysfunction, cell damage, and cell apoptosis [21].

Superoxide anion (O_2_^•−^) is considered to be one of the most important ROS, and the production of O_2_^•−^ in the mitochondrial matrix depends on O_2_ concentration, proton motive force, and the NADH/NAD+ ratios [22, 23]. Under the action of NADPH, oxygen can produce O_2_^•−^, which reacts with NO to produce peroxynitrite anion (ONOO−). Besides, NO can also further accelerate the generation of O_2_^•−^ through mitochondrial electron transport chain (ETC) [24]. Additionally, under the action of superoxide dismutase (SOD), two molecules of O_2_^•−^ and one molecule of water generate H_2_O_2_, which obtains electrons from ferrous ions to generate hydroxyl radical (HO•) [25].

The massive accumulation of mitochondrial succinate is believed to be the key cause of IRI damage. Hypoxia leads to the accumulation of succinate and its metabolites. A large amount of succinate accumulated in the reperfusion phase is oxidized by complex II and further transfers the generated electrons to complex I. This process is called reverse electron transport (RET) and is considered to be the main source of ROS generation [26–29]. Both succinate dehydrogenase inhibitor dimethyl malonate and mitochondrial complex I inhibitor, rotenone, could reduce the content of ROS to play a protective role in IRI. Importantly, mitochondrial complexes I to IV produce proton gradients through the mitochondrial inner membrane, and complex V converts ADP into adenosine triphosphate (ATP), which is the main energy source of cells [30]. Some studies have shown that some substances affect the process of IRI; for instance, heterodimer hypoxia-inducible factor 1 alpha (HIF-1*α*) is a molecular sensor that regulates the transcription of various downstream genes by ischemic signals, which is involved in various energy metabolism and apoptosis [31]. Propofol (but not sevoflurane) inhibits mitochondrial dysfunction by protecting the oxidative respiratory chains and mitochondrial membrane potential which limit ATP consumption in the liver by regulating complex V and also protect mitochondrial function by limiting HIF-1*α* activation and decreasing oxidative stress [32].

**Figure 1:**
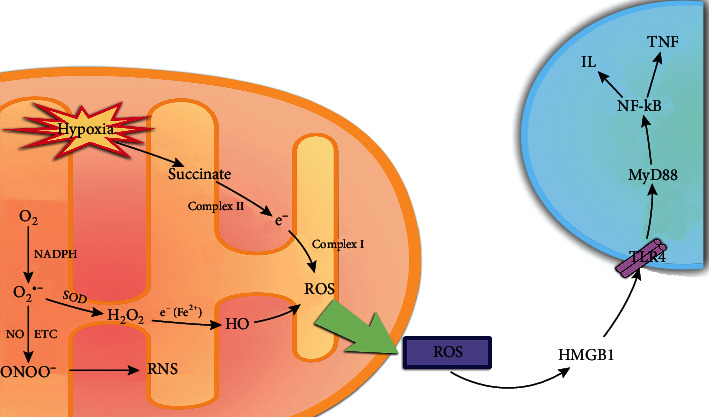
The arrows refer to the role of promotion, and the symbol of “┴” refers to the role of inhibition. Under the action of NADPH, O_2_ can produce O_2_^•−^, which is considered to be one of the most important ROS. On the one hand, O_2_^•−^ can react with super NO to produce ONOO^−^. On the other hand, two molecules of O_2_^•−^ can generate H_2_O_2_ and one molecule of water under the action of SOD. The H_2_O_2_ obtains electrons from ferrous ions to generate HO. In addition, hypoxia leads to the accumulation of succinate and its metabolites. A large amount of accumulated succinate is oxidized by complex II and further transmits electrons to complex I and generates a large amount of ROS. Further, ROS passes through HMGB1-TLR1-MyD88-NF-*κ*B signaling pathway stimulates the release of inflammatory factors and induces cell apoptosis.

Mitochondria produce a large amount of ROS in the ischemic phase. When reperfusion occurs, ROS can induce neutrophils to accumulate in the liver and further lead to cell damage and inflammatory response. Damaged hepatocytes release damage-associated molecular patterns (DAMPs), such as high mobility group box 1 (HMGB1) and histones, which promotes the generation of the neutrophil extracellular trap through Toll-like receptor 4- (TLR4-) myeloid differentiation primary response gene 88 (MyD88) and TLR9-MyD88 to aggravate IRI and induce KCs to secrete cytokines. Besides, TLRs are one of the most representative receptors in innate immunity, which are recognized and combined with the DAMPs to regulate the MyD88-dependent signaling pathway, stimulating the release of proinflammatory cytokines and inducing apoptosis by activating nuclear factor *κ*B (NF-*κ*B) [33–36]. DAMPs and ROS activated NF-*κ*B in the immune response, which released various proinflammatory cytokines, including interleukin 1*α* (IL-1*α*), IL-1*β*, IL-2, IL-3, IL-6, IL-8, and tumor necrosis factor-alpha (TNF-*α*). TLR4 in KCs receptors is activated by HMGB1. Subsequently, KCs release ROS to increase the number of CD4^+^ T cells in the extracellular matrix (ECM), leading to cell damage and further production of ROS [37]. Inhibition of the necrotic pathways may slow down the tissue damage induced by HMGB1 and inflammation-mediated diseases such as sepsis, hepatitis, and IRI [38] (Figure 1).

## 3. Mitochondrial Fission

Mitochondria are bilayer membrane organelles, which shape and size of the mitochondria are different in different cells, and mitochondria are uniformly spherical or ovoid in liver cells. Besides, mitochondria are also highly dynamic organelles that adapt to stress through various pathways, such as mitochondria have proteolytic systems to degrade their misfolded proteins. Damaged mitochondrial outer membrane proteins could be degraded by proteasomes or be cleared by mitochondrial autophagy. Mitochondria sustain the integrity of structure and function through continuous fusion and fission [39, 40].

Mitochondrial quality control (QC) is essential for regulating mitochondrial balance, which can be achieved through a series of processes such as mitochondrial fusion, fission, and selective mitochondrial autophagy [41–43]. In mammals, mitochondrial homeostasis and mitochondrial autophagy are essential for IRI, and mitochondrial fusion and fission can isolate damaged mitochondria and maintain the relative balance of mitochondrial components, such as DNA, protein, and metabolites, and mitochondrial autophagy is responsible for the degradation and recycling of damaged mitochondria [44–46].

Mitochondrial fusion and fission are all regulated by dynamin-related proteins (DRPs). There are four receptors for dynamin family members on mitochondria: mitochondrial dynamic proteins of 49 and 51 kDa, mitochondrial fission protein 1 (Fis1), and mitochondrial fission factor (Mff), which have great significance in mitochondrial QC [47, 48]. In IRI, decreased intracellular ATP concentration can induce mitochondrial fission to maintain the number of mitochondria by upregulating the expression of dynamin-related protein 1 (Drp1) and fission 1 (Fis1), while excessive mitochondrial fission will cause mitochondrial fragmentation and active cell apoptosis. Therefore, for the specific cell signaling pathway mediated by Drp1, a variety of substances have been discovered or designed to slow down excessive mitochondrial damage and IRI. Among them, exogenous application of irisin can significantly inhibit the expression of Drp1 and Fis1 to play a protective role [18].

**Figure 2:**
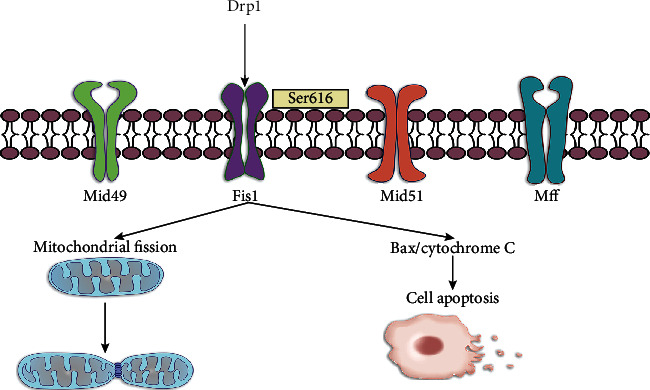
The arrows refer to the role of promotion, and the symbol of “┴” refers to the role of inhibition. Under normal circumstances, mitochondria contain the four receptors of Drp1 (Mid49, Mid51, Fis1, and Mff). The phosphorylation of Drp1 at Ser-616 causes mitochondrial fission and hepatocyte apoptosis.

CDK1/cyclin B complex can stimulate the phosphorylation of Drp1 at Ser-616 to cause mitochondrial fission and hepatocyte apoptosis, while liver stimulator substance (HSS) can significantly inhibit the expression of cyclin-dependent kinase 1 (CDK1) and Bax, reduce Drp1 phosphorylation on Ser-616, and release of cytochrome C to resist IRI [49]. Some studies have shown that HSS protects the liver from various toxins, such as carbon tetrachloride, D-galactosamine, ethanol, and hydrogen peroxide (H_2_O_2_), which could inhibit cytochrome C leakage and the activity of caspase to protect liver cells [50]. Augmenter of liver regeneration (ALR) can inhibit the phosphorylation of Drp1 to play a protective role, while small ubiquitin-like modification (SUMOylation) can promote Drp1 activation to accelerate mitochondrial division. Further research results show that ALR can inhibit Drp1 SUMOylation to resist the role of IRI [51] (Figure 2).

## 4. mPTP and Mitophagy

Under normal circumstances, as the main energy source, ATP is produced by mitochondria with a complete structure and function. In the ischemic phase, mitochondria are damaged in a large amount, and ATP is consumed in large quantities; thus, cellular respiration is converted from aerobic to anaerobic, which activates Na^+^/H^+^ antiporter to increase the concentration of sodium, and further increases the intracellular calcium to activate the apoptosis pathways [21].

Importantly, autophagy is an important metabolic process of cells, and its imbalance is related to various pathological processes, such as inflammation and oxidative stress. Autophagy is an important metabolic process in cells, and its imbalance is related to a variety of pathological processes, such as inflammation and oxidative stress. Sirtuin 1 (SIRT1), as a NAD+-dependent type III protein deacetylase, can regulate liver lipid metabolism and inflammation, strengthen autophagy, and restore the number of mitochondria and membrane potential [52]. The overexpression of SIRT1 significantly inhibits the occurrence of abnormal mitochondrial autophagy and permeability transition, and its function is related to mitofusin-2 (MFN2), which locates on the outer membrane of mitochondria and participates in mitochondrial fusion and fission to maintain mitochondrial stability [53, 54]. SIRT1 activates autophagy via various signaling pathways, and overexpression of SIRT1 induces the deacetylation of MFN2 to reverse various pathological processes [55]. MFN2 also downregulates mitochondrial Ca^2+^ uptake 1/2 to reduce calcium influx and inhibit apoptosis. Therefore, lacking SIRT1 induces autophagy deficiency and mitochondrial dysfunction [56].

The uptake of calcium in the liver's mitochondria is via mitochondrial calcium uniporter complex (MCUC) and mPTP, which accumulates large amounts of calcium in the mitochondria during ischemia, and it mediates the occurrence of mPTP during reoxygenation, leading to apoptosis [57]. Present researches have shown that some proteins are involved in calcium uptake in mitochondria, such as MCUC, leucine zipper and EF-hand-containing transmembrane protein 1 (Letm1), mitochondrial ryanodine receptor type 1, and uncoupling proteins [58]. Once mPTP is formed, small molecule substances will diffuse to the mitochondrial inner membranes, inducing mitochondrial depolarization, uncoupling, and swelling, leading to ATP consumption, necrosis, and apoptosis [59]. Although many mechanisms are unknown during mPTP formation, some proteins have been proved to play a role in this process, such as ATP synthase, adenine nucleotide translocase, and cyclophilin D (CypD). Among them, CypD can ectopic to the inner mitochondrial membrane to activate mPTP to promote cell death, and CypD, as the main risk factor of mPTP, can be regulated by the pore inhibitor Cyclosporin A (CsA) [60].

**Figure 3:**
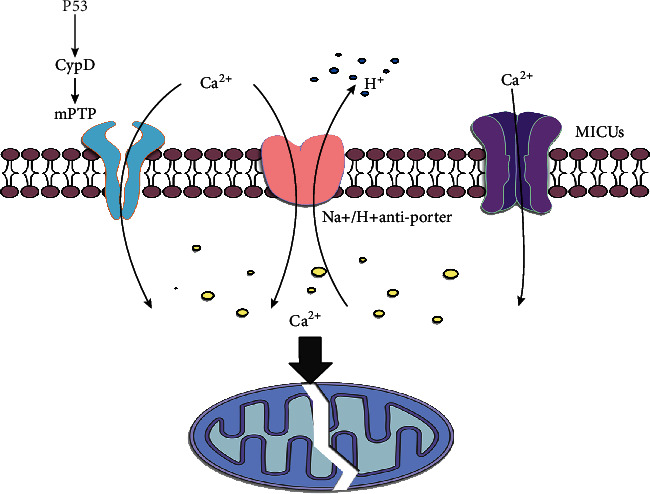
The arrows refer to the role of promotion, and the symbol of “┴” refers to the role of inhibition. Na+/H+ antiporter and mitochondrial Ca2+ uptake 1/2 (MICUs), and the P53-CypD signaling pathway is an important regulatory pathway for mPTP formation and calcium overload. Excessive calcium concentration in mitochondria can induce mitochondrial damage and apoptosis.

For the CypD signaling pathway, various substances have been designed or discovered to inhibit CypD-mediated mitochondrial apoptosis. Tumor suppressor p53 moves to mitochondria and combines with CypD to promote mPTP formation, while similar to the effect of CypD knockout, decreasing the production of spastic paraplegia 7 (SPG7) causes an increase in calcium content. Thus, silencing SPG7-CypD binding prevents calcium and ROS-mediated cell death [61]. Some research results show that Ginsenoside, the main component of ginseng, can significantly inhibit the expression of Caspase-3, Caspase-9, and cytochrome C to inhibit cell apoptosis. Further experiments have confirmed that Ginsenoside can slow down mitochondrial injury and IRI by inhibiting the CypD pathway [62], and in mouse and human liver tissues, C31, a small molecule inhibitor of CypD, has a high affinity for CypD, which can reduce calcium ion-induced mitochondrial swelling and destruction by blocking the opening of mPTP to play a role in protecting the liver from IRI [19]. Although rhein can induce the opening of mPTP to cause necrosis and apoptosis of hepatocytes, CsA can combine with CypD to reduce the toxic effect of rhein by inhibiting the opening of mPTP [63]. Besides, DS44170716, a small-molecule compound, has a significant protective effect on calcium-mediated mitochondrial swelling, and its protective effect is similar to that of CsA. Through further research, it is found that DS44170716 can block calcium overload by inhibiting mitochondrial complexes III, IV, and V, which can be used as a new mechanism of action against calcium ion-induced apoptosis [64] (Figure 3).

## 5. Protective Mechanisms of IRI

### 5.1. Functional Proteins

Previous studies have shown that there are some antioxidant resistance systems in the body, including SOD, thioredoxins, glutathione peroxidase (GPx), glutathione (GSH), mitochondrial iron chelator ferritin, and vitamin E. Reducing the content of antioxidants, for instance, Manganese SOD and catalase could aggravate IRI [65]. Besides, mitochondrial NADP^+^-dependent isocitrate dehydrogenase 2 (IDH2) is a major regulator of NADPH. IRI could cause IDH2 dysfunction to reduce the level of NADPH and GSH-related mitochondrial antioxidant systems GPx and GSH reductase [66]. As the main risk factor of liver IRI, the production and consumption of ROS are kept in a dynamic equilibrium range, and the mitochondrial oxidative respiratory chain in the inner membrane of mitochondria as the main source of ROS contains a large number of enzymes that can replace oxygen with O_2_^•−^ and H_2_O_2_ [67]. As a kind of protective mechanism, cells activate antioxidant enzymes such as catalase, GSH S-transferase (GST), thioredoxin 2, SOD, GPx, heme oxygenase 1 (HO-1), and NAD (P) H to remove excessive ROS [68].

On the other hand, peroxisome proliferator-activated receptor-*γ* (PPAR*γ*) was continuously activated with local liver ischemia for 90 minutes and reperfusion for 8 hours in male mice. To determine the effect of PPAR*γ*, some researchers used PPAR*γ* agonist rosiglitazone and connecting peptide to treat mice, which decreased the occurrence of IRI [69]. PPAR*γ* has a protective effect on IRI, which increases the level of SOD and H_2_O_2_ hydrolase, decreases the level of nicotinamide adenine dinucleotide phosphate oxidase, slows down the degradation of Bcl-2 and Bcl-xl, and significantly reduces the phosphorylation level of AKT to inhibit p65 nuclear translocation and inflammatory response [70–72]. Besides, peroxisome proliferator-activated receptor *γ* coactivator 1*α* (PGC-1*α*) is a major regulator of mitochondria and ROS, which promotes the release of a series of antioxidant enzymes to resist oxidative injury [73]. The increase in the number of peroxisomes activates PGC-1*α*, a protein involved in mitochondrial biosynthesis, which can stimulate mitochondrial production and increase the content of ROS enzymes, such as manganese superoxide dismutase 2 and the uncoupling protein 2, to remove excess ROS [22].

Mitochondrial dysfunction is an important cause of IRI, owing to a large amount of ROS production, which leads to the transition of mitochondrial permeability and aggravates mitochondrial damage. In recent years, the mechanisms of melatonin have been gradually revealed, and more and more studies have shown that melatonin pretreatment can resist IRI by inhibiting the formation of mPTP and affect the expression of some markers, such as inflammation (TNF-*α*/NF-*κ*B/IL-1*β*/mmp-9), oxidative stress (NOX-1/NOX-2), apoptosis (Caspase-3/Bax), and mitochondrial damage (cytochrome C) [74, 75]. It is generally considered that IRI is closely related to mitochondrial ROS production, while melatonin inhibits ROS-induced oxidation of proteins, lipids, and DNA. As a powerful free radical scavenger, melatonin endogenously produces a large amount of indoleamine, and it is considered to be a potential antioxidant acts on many pathological processes. Furthermore, melatonin activates the antioxidant enzyme transcription factor signal transducer and activator of transcription 3 (STAT3) by acting on SAFE and JAK2 signaling pathways to remove excessive ROS [76, 77].

Mitochondria are important organelles, which are essential for oxidative stress and cell metabolism. Melatonin protects against mitochondrial dysfunction during IRI, and melatonin and its metabolites regulate a variety of antioxidants and prooxidant enzymes to prevent oxidative stress, which may affect the metabolism of free radicals and the production of corresponding enzymes through the Keap1-Nrf2-ARE pathway. When oxidative stress occurs, Nrf2 is released from Keap1, and then, it is phosphorylated to transcribe into the nucleus to bind to Maf and acts on the antioxidant response element to remove ROS, while melatonin could act on Nrf2 to reduce its degradation [78].

Melatonin reduces mitochondrial swelling and the releases of glutamate dehydrogenase and acts on proteins related to mitochondrial QC, such as Drp1 and MFN2 to directly or indirectly remove free radicals [79]. With further studies, melatonin has been shown to play an active role in various liver diseases, which blocks the expression of VEGF through HIF-1*α* and STAT3 signaling pathways and regulates autophagy and apoptosis of HepG2 cells [80] (Table 1).

### 5.2. Signaling Pathway Proteins

Under physiological conditions, NF-E2-related factor-2 (Nrf2) is considered to be a major regulator of intracellular oxidative balance, as a transcription factor, which combines with its inhibitor Kelch-like ECH-associated protein 1 (Keap1) in the cytoplasm as a combination, while various pathological conditions stimulate the separation of Nrf2 and Keap1, leading to the nuclear translocation of Nrf2 to activate the antioxidant system, such as GSH and NADPH. The large consumption of Nrf2 can increase the susceptibility of cells to toxins [81, 82]. Besides, the transfer of Nrf2 to the nucleus promotes the expression of antioxidant genes such as HO-1, and CDDO-imidazoline (CDDO-Im) as a potential activating factor of Nrf2 exerts an effect on decreasing IRI [83].

Other evidence has shown that monocyte infiltration is the main source of HO-1, in vitro macrophage culture shows that HO-1 can positively regulate the SIRT1 pathway, and HO-1 can inhibit macrophage activation and NF-*κ*B pathway by affecting P53. Therefore, HO-1 deletion can reduce the expression of SIRT1/p53 and accelerate the occurrence of IRI [84, 85]. NF-*κ*B mediates the binding of tumor necrosis factor (TNF) to the corresponding receptor to form a death-inducing signaling complex (DISC), for instance, TNF-associated death domains, Fas-associated death domains, and Caspase-8 to induce apoptosis, while HO-1 attenuates TNF-mediated cells damage [86].

The production of a large amount of ROS can induce inflammation and aggravate IRI through the HMGB1-TLR4-MyD88-NF-*κ*B signaling pathway [33, 34, 87]. Recent studies have shown that IL-17a can accelerate the process of IRI. IL-17a knockout mice and wild mice fed a high-fat diet to form the fatty liver and undergo IRI treatment. The results show that IL-17a knockout mice can significantly slow down liver damage caused by IRI, and this specific mechanism is related to the inhibition of the IL-17a/NF-*κ*B signaling pathway [88]. IPC can reduce the production of proinflammatory cytokines induced by CD4+, especially IL-2. After IPC, the level of IL-2 is significantly reduced and inhibits the exudation of neutrophils, reduces the production of ROS to limit the content of ROS in the body, and increases the ability of the liver's antioxidant system to reduce inflammation, protect mitochondria, and inhibit cell necrosis and apoptosis [89].

Some other research results show that phosphodiesterase inhibitor cilostazol can promote the expression of Nrf2 and HO-1 to slow down IRI [68, 90, 91] and Brahma-related gene 1 (Brg1) as the core ATPase, whose overexpression increases Nrf2-mediated HO-1 gene transcription to increase antioxidant capacity against liver injury [92]. Another important aspect is the activation of transcription factor 3 (ATF3) inhibits the expression of inflammatory genes in a variety of diseases, which inhibits Nrf2/HO-1 and PI3K/AKT signaling pathways, leading to the activation of TLR4/NF-*κ*B [93].

As another transcription factor, Bach1 encodes HO-1 to degrade serotonin into the free body, carbon monoxide, and bilirubin. Furthermore, microRNA-27a-5p upregulates HO-1 by targeting Bach1 to increase the expression of Bcl-2 and decrease the expression of Caspase-3 [94]. In addition, HO-1 protects the liver by limiting the inflammatory response and enhancing the antiapoptotic pathways; thus, the use of Zinc protoporphyrin (ZnPP) which inhibits the expression of HO-1 could increase the content of cytochrome C and Bax [95], and interferon regulatory factor 9 (IRF9) is considered to be a regulator of IRI, which induces liver apoptosis by reducing the expression of SIRT1 and the level of acetyl-p53 [96]. Furthermore, glucose oxidase (GOX) can mediate the expression of p53 targeting genes Bcl-2 and Bax and promote the release of cytochrome C from mitochondria. CsA can inhibit the entry of P53 into mitochondria to play a protective role [97].

## 6. Future Expectation: Find the New Land

Liver IRI is an important risk factor for liver dysfunction after liver transplantation in patients with fatty liver, nonalcoholic cirrhosis, and hepatocellular carcinoma. Some pathological conditions, such as liver steatosis, are closely related to liver IRI [98]. Many results show that liver steatosis can affect mitochondrial homeostasis during liver transplantation and can reduce the success rate of liver transplantation by increasing the sensitivity of liver tissue to IRI during liver transplantation, which may be related to the formation of mPTP and oxidative stress, but the specific mechanism is still unclear [99]. Therefore, in order to better understand the mechanism of IRI in different diseases and pathological states, we introduce new developments in the field of IRI from two aspects: new technology and new treatment methods.

To begin with, mitochondrial-targeted near-infrared fluorescent probes provide bioimaging and assessment of endogenous ischemia-reperfusion peroxy anion changes. The probe also has deep tissue permeability which could perform real-time imaging of O_2_^•−^ in mouse liver. Of note, probes accurately assess the relationship between O_2_^•−^s and organ damage, and studies have found that IPC and ischemia postconditioning have protective effects on the liver [100]. So far, there are many strategies to deal with IRI, and IPC is the only proven method to improve liver injury. Some researchers prevented IRI by diet restriction on mice, as a result, which reduces liver damage by increasing stress resistance, but the specific mechanism is unclear [101]. Besides, a special structure, MITO-Porter, was previously reported, which is based on the principle that liposomes enter mitochondria through membrane fusion to deliver various therapeutic substances, including antiapoptotic and antioxidant substances. Therefore, MITO-Porter might be a potential target for the treatment of mitochondrial diseases [102].

Furthermore, with a deeper understanding of IRI, some new treatment methods and measures have emerged, such as the use of special drugs or small molecules which act on specific signal pathways to reduce the production of corresponding proteins or inhibit certain key targets. Through reading a lot of literature, it can be found that some new therapeutic targets are getting more and more attention, namely, HO-1, P53, Drp1, SIRT1, and PGC-1*α*. They not only regulate oxidation and antioxidation by affecting specific signaling pathways to reduce the excessive damage of oxidative stress to the body, but also promote the formation of mitochondria to replace the physiological role of damaged mitochondria and inhibit the formation of mPTP to resist the oxidative stress on the mitochondria. Therefore, searching for core signaling pathways and targeted proteins is a key part of IRI treatment. At present, some new research results have shown that the application of proteomics analysis can identify potential therapeutic targets in IRI. Based on the proteomic analysis, the new target proteins may be found, and this may be the key way to treat IRI [103]. Interestingly, some research teams have identified some key determinants of the IRI processes by using integrative “omics” analysis, such as arachidonate 12-lipoxygenase-12-hydroxyeicosatetraenoic acid-G-protein-coupled receptor 31 signaling axis, which is essential for further understanding of IRI [104].

The mechanism of IRI is too complicated, and many have not been discovered and understood, so it needs active exploration to continuously discover new signaling pathways and targets to reveal the specific mechanism of IRI. Of note, this method of regulating gene or protein expression by regulating specific signaling pathways may have huge development in the prospect.

## 7. Conclusion

IRI is common in clinical practice; so far the mechanism of its occurrence has not been fully illuminated, which restricts the level of clinical practice due to insufficient understanding of molecular mechanisms of ischemia-reperfusion. More and more studies have shown that mitochondria are the main source of ROS, and excessive ROS is the main factor of IRI. Some preventive measures and treatment options are emerging, which might inhibit oxidative stress, protect the structure and function of mitochondria, and restrain mPTP formation and calcium overload. All of these are important for alleviating liver IRI and maintaining normal physiological functions of the body.

## Figures and Tables

**Table 1:** Some key target genes or proteins in the process of liver ischemia-reperfusion injury.

Target	Classification	Function	Drug	Refs. (PMID)
ROS	ROS inhibitor	Inhibitor oxidative stress	Rotenone	22706634
			SPG7	26387735
			HO-1	28842295
			Melatonin	26993080
Drp1	Drp1 agonist	Promotes mitochondrial fission and apoptosis	ALR	33110216
	Drp1 inhibitor	Inhibits mitochondrial fission and apoptosis	HSS	28646508
		Inhibits mitochondrial fission and apoptosis	Irisin	30388684
P53	P53 agonist	Promotes mitochondrial fission and apoptosis	GOX	26884717
	P53 inhibitor	Inhibits the formation of mPTP	CsA	30496631
			C31	31336123
			SPG7	26387735
MFN2	MFN2 agonist	Inhibits abnormal mitophagy	SIRT1	26184910
Nrf2	Nrf2 agonist	Inhibits oxidative stress	Cilostazol	25951827
			Brg1	28569786
	Nrf2 inhibitor	Promotes oxidative stress	ATF	25359217
HO-1	HO-1 agonist	Inhibits mitochondrial fission and apoptosis	Bach1	30145824
	HO-1 inhibitor	Promotes mitochondrial fission and apoptosis	ZnPP	25319231

## Abbreviations

ATP: Adenosine triphosphate
CsA: Cyclosporin A
Cyp: Cyclophilin
DAMPs: Damage-associated molecular patterns
Drp1: Dynamin-related protein 1
ETC: Electron transport chain
GSH: Glutathione
GPx: Glutathione peroxidase
HO-1: Heme oxygenase 1
HSS: Hepatic stimulator substance
HIF-1*α*: Heterodimer hypoxia-inducible factor 1 alpha
H_2_O_2_: Hydrogen peroxide
IL: Interleukin
IRI: Ischemia-reperfusion injury
IPC: Ischemic preconditioning
IDH2: Isocitrate dehydrogenase 2
Keap1: Kelch-like ECH-associated protein 1
KCs: Kupffer cells
Letm1: Leucine zipper and EF-hand-containing transmembrane protein 1
MCUC: Mitochondrial Ca^2+^ uniporter complex
Mff: Mitochondrial fission factor
Fis1: Mitochondrial fission protein 1
mPTPs: Mitochondrial membrane permeable transport pores
MFN2: Mitofusin-2
MyD88: Myeloid differentiation primary response gene 88
Nrf2: NF-E2-related factor-2
NF-*κ*B: Nuclear factor *κ*B
PGC-1*α*: Peroxisome proliferator-activated receptor *γ* coactivator 1*α*
PPAR*γ*: Peroxisome proliferator-activated receptor-*γ*
ONOO−: Peroxynitrite anion
ROS: Reactive oxygen species
QC: Quality control
RET: Reverse electron transport
STAT3: Signal transducer and activator of transcription 3
SIRT1: Silent information regulators (sirtuins)
O_2_^•−^: Superoxide anion
SOD: Superoxide dismutase
TNF-*α*: Tumor necrosis factor-alpha
12-HETE: 12-Hydroxyeicosatetraenoic acid.
